# Longitudinal Monitoring of Clinician-Patient Video Visits During the Peak of the COVID-19 Pandemic: Adoption and Sustained Challenges in an Integrated Health Care Delivery System

**DOI:** 10.2196/54008

**Published:** 2024-04-08

**Authors:** Jessica A Palakshappa, Erica R Hale, Joshua D Brown, Carol A Kittel, Emily Dressler, Gary E Rosenthal, Sarah L Cutrona, Kristie L Foley, Emily R Haines, Thomas K Houston II

**Affiliations:** 1 Atrium Health Wake Forest Baptist Winston Salem, NC United States; 2 Wake Forest University School of Medicine Winston Salem, NC United States; 3 Department of Population and Quantitative Health Sciences University of Massachusetts Chan Medical School Worcester, MA United States; 4 Center for Healthcare Organization and Implementation Research Veterans Affairs Bedford Healthcare System Bedford, MA United States

**Keywords:** telehealth, telemedicine, e-health, eHealth, video visits, video, ICT, information and communication technology, survey, surveys, adoption, usability, experience, experiences, attitude, attitudes, opinion, perception, perceptions, perspective, perspectives, COVID-19

## Abstract

**Background:**

Numerous prior opinion papers, administrative electronic health record data studies, and cross-sectional surveys of telehealth during the pandemic have been published, but none have combined assessments of video visit success monitoring with longitudinal assessments of perceived challenges to the rapid adoption of video visits during the pandemic.

**Objective:**

This study aims to quantify (1) the use of video visits (compared with in-person and telephone visits) over time during the pandemic, (2) video visit successful connection rates, and (3) changes in perceived video visit challenges.

**Methods:**

A web-based survey was developed for the dual purpose of monitoring and improving video visit implementation in our health care system during the COVID-19 pandemic. The survey included questions regarding rates of in-person, telephone, and video visits for clinician-patient encounters; the rate of successful connection for video visits; and perceived challenges to video visits (eg, software, hardware, bandwidth, and technology literacy). The survey was distributed via email to physicians, advanced practice professionals, and clinicians in May 2020. The survey was repeated in March 2021. Differences between the 2020 and 2021 responses were adjusted for within-respondent correlation across surveys and tested using generalized estimating equations.

**Results:**

A total of 1126 surveys were completed (511 surveys in 2020 and 615 surveys in 2021). In 2020, only 21.7% (73/336) of clinicians reported no difficulty connecting with patients during video visits and 28.6% (93/325) of clinicians reported no difficulty in 2021. The distribution of the percentage of successfully connected video visits (“Over the past two weeks of scheduled visits, what percentage did you successfully connect with patients by video?”) was not significantly different between 2020 and 2021 (*P*=.74). Challenges in conducting video visits persisted over time. Poor connectivity was the most common challenge reported by clinicians. This response increased over time, with 30.5% (156/511) selecting it as a challenge in 2020 and 37.1% (228/615) in 2021 (*P*=.01). Patients not having access to their electronic health record portals was also a commonly reported challenge (109/511, 21.3% in 2020 and 137/615, 22.3% in 2021, *P*=.73).

**Conclusions:**

During the pandemic, our health care delivery system rapidly adopted synchronous patient-clinician communication using video visits. As experience with video visits increased, the reported failure rate did not significantly decline, and clinicians continued to report challenges related to general network connectivity and patient access to technology.

## Introduction

Interest in telehealth from policy makers, health care providers, patients, and families continues to grow [1], including newer modalities such as video visits [2]. Video visits refer to clinician-patient communication that includes real time video and audio assessment of the patient when the clinician is in a different location. Video visits have the potential to improve efficiency for clinicians and to improve access for patients, particularly those who reside in rural areas or with transportation barriers [3]. The technology to support video visits has existed for decades; however, only a small minority of clinicians used this form of telehealth in their practices [4] prior to the COVID-19 pandemic. There are multiple reasons why telehealth adoption was slow despite its potential benefits including changing cost and reimbursement policies, federal and state licensing laws, incompatible electronic health records, and gaps in internet access in certain areas.

The COVID-19 pandemic and subsequent public health emergency led to fundamental shifts in how health care was delivered in the United States, including the rapid adoption of telehealth services. Before the public health emergency, approximately 13,000 fee-for-service Medicare beneficiaries received telehealth services in a week and that number increased to nearly 1.7 million beneficiaries by the last week of April 2020 [5]. While the need to avoid in-person contact fueled the initial rapid rise, regulations and restrictions were temporarily lifted during this time facilitating its use. Clinicians were also paid for telehealth services at the same rate as in-person medical services. Several studies have reported on the rapid uptake of telehealth, including video visits, in this context [6-8]. However, few reports have explored rates of success and failure of video visits over time. The challenges clinicians face in conducting video visits have also not been explored. Understanding these challenges will be important for improving and expanding the reach of telehealth services after the pandemic has ended.

In the context of the rapidly increasing use of telehealth to conduct video visits, and consistent with the sociotechnical model’s [9] emphasis on monitoring the implementation of health information technology in complex adaptive health care systems, our health care delivery system initiated a series of brief assessments of video visit adoption. The research objective of this report is to summarize the findings of the video visit monitoring including (1) the use of video visits (compared with in-person and telephone visits) over time during the pandemic, (2) video visit successful connection rates, and (3) changes in perceived video visit challenges. With patient and clinician skills and experience with video visits increasing over time, our primary hypothesis was that clinicians’ perceived challenges to completing video visits (eg, software, hardware, bandwidth, and technology literacy) would decline over time.

## Methods

### Study Design

The design was a longitudinal series of 2 cross-sectional assessments (2020 and 2021). In summary, for the dual purpose of monitoring and improving telehealth implementation in our health care delivery system, institutional leaders developed a brief web-based survey regarding the use of video visits and challenges. The survey was initially distributed in 2020. Given the ongoing public health emergency and the need to re-evaluate telehealth use, the survey was repeated in 2021. Institutional leaders encouraged clinicians to complete the survey, communicating encouragement via emails and announcements at in-person faculty and departmental meetings.

### Ethical Considerations

As the brief assessments were distributed by the clinical system as part of ongoing quality improvement, the project was approved as an research protocol as exempt from human participants approval by the Wake Forest University School of Medicine Institutional Review Board (IRB00077473). The survey did not collect identifying information from the participants.

### Survey Development

Published in 2010 by Sittig and Singh [9], the sociotechnical model of health information technology was the first to fully emphasize the importance of system monitoring in implementation frameworks. Key aspects of monitoring, including measuring how the technology is being used by clinicians and whether implementation outcomes are being achieved, were considered when developing the survey. The survey was developed with a literature search, expert review, and iterative pilot-testing (see Multimedia Appendix 1). The final survey included 12 questions related to rates of in-person, telephone, and video for clinician-patient encounters; the rate of successful connection for video visits; and perceived challenges to video visits (eg, software, hardware, bandwidth, and technology literacy).

### Study Population

The study population included all outpatient clinicians practicing across the health care delivery system; we excluded clinicians without direct patient care responsibilities. The system includes 5 hospitals and over 350 primary care and specialty clinics that provide care to over 2 million persons annually. The brief assessment was distributed to clinicians in 2020 and 2021 (1937 clinicians and 2843 clinicians, respectively).

### Survey Distribution and Data Collection

As we are an integrated health care delivery system, we had access to the contact details of all providers. Our group practice clinical operations executive committee facilitated the survey distribution by requesting that each department chair and clinical service line director send an email to their team of providers to notify them of the survey and encourage completion. Surveys were collected and managed using REDCap (Research Electronic Data Capture), a secure, web-based app designed to support data capture [10,11]. A unique survey link was distributed via email to each clinician in May 2020 and March 2021. The system sent up to 2 reminder emails if the recipient had not yet completed the survey.

### Statistical Analysis

To take full advantage of the data collected, we first analyzed the results as 2 cross-sectional surveys. In this primary analysis, we included all respondents in each year. We recognize that a subset of clinicians also responded in both years. Thus, as a secondary analysis, we analyzed the data limited to the longitudinal cohort who participated in both years. First, summary statistics are presented as count (frequency) for categorical variables and mean (SD) or median (IQR) for continuous variables as appropriate. Generalized estimating equations were then used to model frequency distributions of in-person, telephone, and video visits, and patient and clinician challenges. These logit models were adjusted for within-respondent correlation across surveys via an exchangeable correlation structure. *P* values of .05 were considered statistically significant. *P* values for multiple comparisons in frequency distributions of in-person, telephone, and video visits between physicians, advanced practice professionals (APPs), and other clinicians were adjusted via the Tukey-Kramer method to control for type I errors with a corrected *P* value <.05 deemed statistically significant [12]. All statistical analyses were performed with R (version 4.2.1; R Core Team) [13].

We recognize that a subset of clinicians responded in both years. Thus, as a secondary analysis, we analyzed the data limited to the longitudinal cohort who participated in both surveys. For the secondary analysis, matched pairs analyses were performed as were performed in the entire sample with only those responses from clinicians that completed both surveys.

## Results

### Surveillance Participation and Participant Characteristics

In 2020, 1937 surveys were sent and 511 responses were received (response rate 26.4%). In 2021, 2843 surveys were sent and 615 responses were received (response rate 21.6%). In both years, over 55% of the respondents were physicians from a wide range of clinical specialties. About half of the clinicians who completed the survey in 2020 also completed it in 2021 (Table 1).

**Table 1:** Characteristics of health care clinicians.

			Response rate in 2020, n (%)		Response rate in 2021, n (%)
**Clinician type^a^**					
	Physician	300 (58.7)		353 (57.4)	
	Physician assistant or nurse practitioner	195 (38.2)		216 (35.1)	
	Other allied health professionals	16 (3.1)		46 (7.5)	
**Physician specialties^b^**					
	Internal medical subspecialty	84 (28)		68 (19.3)	
	General internal medicine and geriatrics	46 (15.3)		43 (12.2)	
	Family medicine	34 (11.3)		42 (11.9)	
	Pediatrics (general)	27 (9)		45 (12.7)	
	Surgery subspecialties	26 (8.7)		34 (9.6)	
	Pain medicine, anesthesia, and rehabilitation	25 (8.3)		18 (5.1)	
	Pediatric subspecialties	20 (6.7)		16 (4.5)	
	Neurology	19 (6.3)		14 (4)	
	Psychiatry and psychology	9 (3)		42 (11.9)	
	Obstetrics and gynecology	7 (2.3)		22 (6.2)	
	Others	3 (1)		9 (2.5)	

^a^n=511 responses in 2020 and n=615 responses in 2021.

^b^n=300 responses in 2020 and n=353 responses in 2021.

### Health Care Delivery by In-Person and Telephone

To place the volume of telehealth in context, we first asked about the number of in-person encounters completed over the past 2 weeks (Table 2). The distribution of responses differed between 2020 and 2021 (*P*<.001). Modeled probabilities show fewer respondents reported zero (22.2% vs 4.9%) or 1 to 10 (33.5% vs 11.5%) in-person visits in 2021 as compared with 2020. The volume of in-person visits increased over time (Table 2). Further, the majority of respondents (399/509, 78.4%) reported at least 1 telephone visit in 2020 and 65.7% (369/562) in 2021 although the distribution of responses differed from 2020 to 2021 (*P*<.001).

**Table 2:** Health care delivery (through in-person visits, telephone visits, and video visits) reported by providers in 2020 and 2021.

Over the past 2 weeks, thinking of your outpatient clinics, please approximate how many of your patient encounters were scheduled as follows		Health care delivery in 2020 (N=511), n (%)	Health care delivery in 2021 (N=615), n (%)	*P* value^a^	
**In-person visits**					<.001
	0	113 (22.2)	28 (4.9)		
	1 to 10	170 (33.5)	65 (11.5)		
	11 to 25	105 (20.7)	132 (23.3)		
	26 to 50	69 (13.6)	139 (24.6)		
	Over 50	51 (10)	202 (35.7)		
**Telephone visits**					<.001
	0	110 (21.6)	193 (34.3)		
	1 to 10	230 (45.2)	316 (56.2)		
	11 to 25	110 (21.6)	44 (8)		
	26 to 50	37 (7.3)	6 (1.1)		
	Over 50	22 (4.3)	2 (0.4)		
**Video visits**					<.001
	0	174 (34.1)	239 (42.4)		
	1 to 10	199 (39)	260 (46.1)		
	11 to 25	95 (18.6)	38 (6.7)		
	26 to 50	30 (5.9)	16 (2.8)		
	Over 50	12 (2.4)	11 (2)		

^a^Differences in frequency distributions between 2020 and 2021 tested via generalized estimating equation modeling; *P* value adjusted using Tukey-Kramer method to control for type I errors.

### Health Care Delivery by Video Visits

Many health care providers were engaged in virtual care, with 65.9% (336/510) health care providers reporting video visit encounters in 2020 and 57.6% (325/564) health care providers reporting video visit encounters in 2021 (Table 2) although the distribution of responses again changed from 2020 to 2021 (*P*<.001). Compared with 2020, fewer 2021 respondents reported 11-25 (18.6% vs 6.7%), 26-50 (5.9% vs 2.8%), or over 50 (2.4% vs 2%) visits.

Secondary analyses were robust to missing data and showed that the results (distributions of in-person, phone, and video visits) did not change when limiting the data to only respondents who participated in both surveys.

### Comparing Health Care Delivery by Physicians, APPs, and Others

We also compared health care delivery modality by type of clinician (physicians, APPs, or others). Patterns of health care delivery reported in the overall sample were similar in the physician, APP, and other subgroups. There were no significant differences between physicians and APPs in the number of patient encounters that were completed as in-person, telephone visits, or video visits in 2020 or 2021.

### Perceived Challenges to Patient-Clinician Connection Using Video Visits

**Figure 1:**
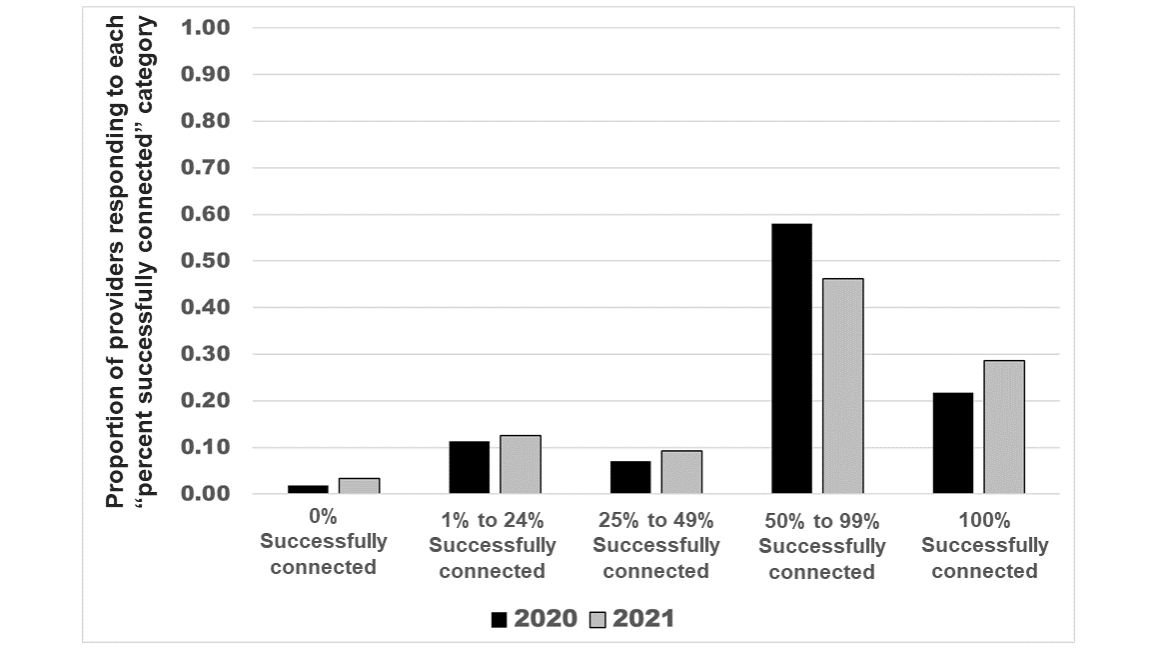
Percentage of successful provider and patient connection in the scheduled video visits, by comparing 2020 and 2021 results. Survey Question: “Over the past two weeks, of the scheduled video visits, what percentage did you successfully connect with the patients by video?” Comparing 2020 versus 2021, no significant difference (*P*=.74); the *P* value was adjusted using the Tukey-Kramer method to control for type I errors.

The use of video visits came with challenges. In 2020, only 21.7% (73/336) of clinicians reported no difficulty connecting with patients during video visits and 28.6% (93/325) of clinicians reported no difficulty in 2021 (Figure 1). The distribution of the percentage of successfully connected video visits (“Over the past two weeks of scheduled visits, what percentage did you successfully connect with patients by video?”) was not significantly different between 2020 and 2021 (*P*=.74, Figure 1). There was also no significant difference between physicians and APPs in the rate of successful video connection with patients in either year.

Clinicians were asked about the challenges in successfully completing video visits (see Table 3). The most commonly reported challenge was poor connectivity. This response increased over time with 30.5% (156/511) selecting it as a challenge in 2020 and 37.1% (228/615) selecting it as a challenge in 2021 (*P*=.01). Patients not having access to their electronic health record portals was also a commonly reported challenge (109/511, 21.3% in 2020 and 137/615, 22.3% in 2021; *P*=.73).

**Table 3:** Clinician-reported challenges encountered on scheduled clinical video telehealth during the COVID-19 pandemic.

If you had challenges with successfully completing video visits with patients, what were the reasons for the challenges (check all that apply)?		Challenges encountered in 2020 (N=511), n (%)	Challenges encountered in 2021 (N=615), n (%)	*P* value^a^
**Patient challenges**				
	The patient did not have access to a smartphone or computer with camera.	135 (26.4)	179 (29.1)	.28
	The patient did not have patient portal access.^b^	109 (21.3)	137 (22.3)	.73
	The patient did not have access to the internet.	73 (14.3)	112 (18.2)	.08
	The patient did not have the needed software.	69 (13.5)	74 (12)	.45
**Clinician challenges**				
	There was a problem with network connectivity (poor connectivity).	156 (30.5)	228 (37.1)	.008
	There was a problem with the clinician software.	54 (10.6)	55 (8.9)	.34
	There was a problem with clinician hardware.	28 (5.5)	30 (4.9)	.63

^a^*P* value adjusted using the Tukey-Kramer method to control for type I errors.

^b^For some clinical video visits, a prerequisite was that patients needed to have registered with the patient portal.

## Discussion

### Principal Findings

Although telehealth technology was available in our health care delivery system prior to the COVID-19 pandemic, it saw only limited use for providing synchronous care to patients prior to the pandemic. Inconsistent reimbursement for services, restrictions on the physical location of patients and clinicians during telehealth, and rules about types of visits that were acceptable for telehealth services all contributed to its limited use [14,15]. With the pandemic, and consistent with other reports, our health care delivery system rapidly expanded the provision of clinical care by way of video visits. Later in the pandemic, in-person visits did increase, but the use of video visits remained well above prepandemic levels.

Overall, there has been a shift toward a more positive sentiment about telehealth and telemedicine since the start of the pandemic. A scoping review by Doraiswamy et al [16] reported 543 telehealth-related papers (mostly opinions, commentaries, and perspectives; 61%) published across 331 different journals from January to June 2020. Most of these new reports had a “celebratory” or favorable sentiment about the use of telehealth. The scope of the increase in telehealth during the public health emergency likely contributed to this sentiment though concerns about patient and clinician connection, the lack of physical examinations, and cost-effectiveness were still noted by some. Although our providers reported benefits for clinical video telehealth beyond audio-only calls for patient-provider visits, failure to connect using clinical video visits was common.

While reports have documented challenges with clinical video telehealth [17], few have monitored these challenges over time. During the pandemic, as our health care delivery system’s experience with video visits grew, clinicians did not report a meaningful reduction in connection failure rate. The most frequently reported challenges were general network connectivity and those related to the digital divide (eg, patient lack of internet access, needed software, or internet-connected cameras). Gaps in access to technology and the internet for telehealth may impact some patient groups more than others. For example, older age, rural residence, dual Medicare and Medicaid enrollment, and non-Hispanic Black or Hispanic race or ethnicity have been shown to be associated with a lower probability of technology ownership, access to the internet, and use of the internet for communication in cancer survivors [18]. Further, over 10% of clinicians also reported that they experienced software or hardware challenges (eg, limited availability of internet cameras at a clinical location). Expanding telehealth will require ongoing investments in technology for clinicians. New workflows to support successful connection during video visits and follow-up processes may also be needed.

Our video visit monitoring results were shared with clinical operations leadership. In response to the sustained challenges noted, we initiated a new video visit program to provide patient support prior to scheduled video visits. Our technology navigators are a specially trained, centralized team and are directed to reach out to vulnerable patients and families to facilitate video visit access. A new electronic health record dashboard identified patients with (1) a scheduled video visit and (2) 1 or more risk factors (eg, lack of a prior successful video visit and lack of patient portal access). We further prioritized patients older than 65 years and those living in rural areas. Technology navigators reached out by telephone to contact these at-risk patients to assess their technology access (eg, internet, software, webcam, or smartphone), technology literacy and perceived competence, and availability of at-home support from family and friends. The technology navigators then troubleshoot any challenges noted by the patients and offer to conduct a “practice” video visit. Evaluation of this program is ongoing. In 2022-2023, the navigators contacted 1266 patients at high risk for video visit failure. Among those contacted, 515 requested and were provided assistance. With previsit support from the navigators, the patient-provider scheduled video visit completion rate was 84% as compared with a 60% completion rate among those patients who did not receive support.

Limitations of our video visit surveillance analysis include that the data were collected across 1 health care delivery system with an integrated electronic health care record system. The perceptions and challenges may be different in a smaller health care system and in those with different health care record systems. The survey measured only clinician-reported telehealth use and success rates which may be limited by recall. As with all surveys, our results may be biased as only about one-quarter of the sample responded. It is possible that respondents experienced more challenges conducting video visits than those who did not respond. Further, not all clinicians longitudinally completed both the 2020 and 2021 surveys—due both to response rates and providers leaving and entering the health care system. Thus, changes over time may represent differences in the underlying sample. For example, new clinicians may have been more or less familiar with conducting video visits.

### Conclusions

Recent reviews have noted the need for more evidence related to telehealth’s implementation, effectiveness, and health equity in telehealth access [16,19,20]. Although internet and smartphone access has increased over the last decade (with older adults being one of the fastest-growing subgroups of new adoption), our longitudinal video visit surveillance reveals that the digital divide is still a significant barrier to video visit access.

Although Healthy People 2030 (a set of national objectives to improve health and well-being) includes developmental and research objectives related to patient portals and increasing the use of telehealth to improve access to health services [21], some social determinants of health taxonomies do not include technology access. If telehealth is increasingly an important component of health care access, then technology access (eg, internet, smartphone, patient portal, and connected hardware, such as internet-connected video) should be considered a social determinant of health [22]. A comprehensive solution to overcoming the digital divide has not yet been achieved. However, some partial solutions include directly providing technology to patients, providing detailed instructions, and support services (eg, our technology navigator program), and engaging trusted caregivers (family and friends) who may be able to assist patients [23-27].

## Appendix

Multimedia Appendix 1 Brief Provider Telehealth Assessment.

## Abbreviations

APP: advanced practice professional
REDCap: Research Electronic Data Capture

## References

[1] World Health Organization (2010). Telemedicine: Opportunities and Developments in Member States: Report on the Second Global Survey on eHealth 2009.

[2] Moulaei K, Shanbehzadeh M, Bahaadinbeigy K, Kazemi-Arpanahi H (2022). Survey of the patients' perspectives and preferences in adopting telepharmacy versus in-person visits to the pharmacy: a feasibility study during the COVID-19 pandemic. BMC Med Inform Decis Mak.

[3] Nesbitt TS, Lustig TA (2012). The evolution of telehealth: where have we been and where are we going?. The Role of Telehealth in an Evolving Health Care Environment: Workshop Summary.

[4] Kane CK, Gillis K (2018). The use of telemedicine by physicians: still the exception rather than the rule. Health Aff (Millwood).

[5] Kane Carol K, Gillis Kurt (2018). The use of telemedicine by physicians: still the exception rather than the rule. Health Aff (Millwood).

[6] Weiner JP, Bandeian S, Hatef E, Lans D, Liu A, Lemke KW (2021). In-person and telehealth ambulatory contacts and costs in a large US insured cohort before and during the COVID-19 pandemic. JAMA Netw Open.

[7] Gilson SF, Umscheid CA, Laiteerapong N, Ossey G, Nunes KJ, Shah SD (2020). Growth of ambulatory virtual visits and differential use by patient sociodemographics at one urban academic medical center during the COVID-19 pandemic: retrospective analysis. JMIR Med Inform.

[8] Hatef E, Wilson RF, Hannum SM, Zhang A, Kharrazi H, Weiner JP, Davis SA, Robinson KA (2023). Use of Telehealth During the COVID-19 Era.

[9] Sittig DF, Singh H (2010). A new sociotechnical model for studying health information technology in complex adaptive healthcare systems. Qual Saf Health Care.

[10] Harris PA, Taylor R, Thielke R, Payne J, Gonzalez N, Conde JG (2009). Research electronic data capture (REDCap): a metadata-driven methodology and workflow process for providing translational research informatics support. J Biomed Inform.

[11] Harris PA, Taylor R, Minor BL, Elliott V, Fernandez M, O'Neal L, McLeod L, Delacqua G, Delacqua F, Kirby J, Duda SN (2019). The REDCap consortium: building an international community of software platform partners. J Biomed Inform.

[12] Midway S, Robertson M, Flinn S, Kaller M (2020). Comparing multiple comparisons: practical guidance for choosing the best multiple comparisons test. PeerJ.

[13] R Core Team (2023). R: A Language and Environment for Statistical Computing.

[14] Shaver J (2022). The state of telehealth before and after the COVID-19 pandemic. Prim Care.

[15] Hyder MA, Razzak J (2020). Telemedicine in the United States: an introduction for students and residents. J Med Internet Res.

[16] Doraiswamy S, Abraham A, Mamtani R, Cheema S (2020). Use of telehealth during the COVID-19 pandemic: scoping review. J Med Internet Res.

[17] Garfan S, Alamoodi AH, Zaidan BB, Al-Zobbi M, Hamid RA, Alwan JK, Ahmaro IYY, Khalid ET, Jumaah FM, Albahri OS, Zaidan AA, Albahri AS, Al-Qaysi ZT, Ahmed MA, Shuwandy ML, Salih MM, Zughoul O, Mohammed KI, Momani F (2021). Telehealth utilization during the Covid-19 pandemic: a systematic review. Comput Biol Med.

[18] Lama Y, Davidoff AJ, Vanderpool RC, Jensen RE (2022). Telehealth availability and use of related technologies among medicare-enrolled cancer survivors: cross-sectional findings from the onset of the COVID-19 pandemic. J Med Internet Res.

[19] Moulaei K, Moulaei R, Bahaadinbeigy K (2023). Barriers and facilitators of using health information technologies by women: a scoping review. BMC Med Inform Decis Mak.

[20] Rodriguez JA, Shachar C, Bates DW (2022). Digital inclusion as health care: supporting health care equity with digital-infrastructure initiatives. N Engl J Med.

[21] Healthy people 2030: health IT. U.S. Department of Health and Human Services.

[22] Benda NC, Veinot TC, Sieck CJ, Ancker JS (2020). Broadband internet access is a social determinant of health!. Am J Public Health.

[23] Francis J, Kadylak T, Makki TW, Rikard RV, Cotten SR (2018). Catalyst to connection: when technical difficulties lead to social support for older adults. Am Behav Sci.

[24] Cotten SR, Yost EA, Berkowsky RW, Winstead V, Anderson WA (2016). Designing Technology Training for Older Adults in Continuing Care Retirement Communities, 1st Edition.

[25] Sadasivam RS, Kinney RL, Lemon SC, Shimada SL, Allison JJ, Houston TK (2013). Internet health information seeking is a team sport: analysis of the Pew internet survey. Int J Med Inform.

[26] Luger TM, Hogan TP, Richardson LM, Cioffari-Bailiff L, Harvey K, Houston TK (2016). Older veteran digital disparities: examining the potential for solutions within social networks. J Med Internet Res.

[27] Houston TK, Richardson LM, Cotten SR (2019). Patient-directed digital health technologies: is implementation outpacing evidence?. Med Care.

