# Supplementary material for: Longitudinal Monitoring of Clinician-Patient Video Visits During the Peak of the COVID-19 Pandemic: Adoption and Sustained Challenges in an Integrated Health Care Delivery System
Source: J Med Internet Res. 2024 Apr 8;26:e54008. doi: 10.2196/54008 (PMC11036186; doi:10.2196/54008)
Supplement: Multimedia Appendix 1 [file jmir_v26i1e54008_app1.pdf]

Brief Provider Telehealth Assessment

How is Telehealth Working for You?

We need your help in assessing the current state of Telehealth technology. Your feedback will be used to plan for the future.

Please fill in the assessment below.

Thank you!

The goal of the following 3 questions is to assess the relative rate of your in-person, telephone and video visits over the past 2 weeks.

Over the past two weeks, thinking of your outpatient clinics, please approximate how many of YOUR patient encounters were scheduled as:

	0	1-10	11-25	26-50	over 50
In-person visits?	◯	◯	◯	◯	◯
Telephone visits?	◯	◯	◯	◯	◯
Video visits?	◯	◯	◯	◯	◯

Over the past two weeks, of the scheduled video visits, what percentage did you successfully connect with the patients by video?

◯ 0%
◯ 1-24%
◯ 25-50%
◯ 51-99%
◯ 100%

If you had challenges with successfully completing video visits with patients, what were the reasons for the challenges (check all that apply)?

☐ The patient did not have access to the internet.
☐ The patient did not have access to a smartphone or computer with camera.
☐ The patient did not have the needed software.
☐ The patient did not have MyWakeHealth access.
☐ There was a problem with the provider software.
☐ There was a problem with provider hardware.
☐ There was a problem with network connectivity (poor connectivity).
☐ Patient reported other challenges.
☐ Other (please specify below).

Other

Which of the following hardware have you used while doing a video visit (check all that apply)?

☐ Smartphone
☐ Tablet (iPad or other)
☐ My home desktop or laptop
☐ Work desktop or laptop connected to the WakeHealth network
☐ Work desktop or laptop not connected to the WakeHealth network

Which of the following software have you used while doing a video visit (check all that apply)?

- ☐ EPIC/WakeOne/MyWakeHealth electronic health record integrated Video
- ☐ Doximity Dialer Video
- ☐ WebEx Video
- ☐ Zoom Video
- ☐ Facetime Video
- ☐ Doxy.me Video
- ☐ Other (Please specify below)

Other:

What software for video visits with patients has worked well for you and your patients (check all that apply)?

- ☐ EPIC/WakeOne/MyWakeHealth electronic health record integrated Video
- ☐ Doximity Dialer Video
- ☐ WebEx Video
- ☐ Zoom Video
- ☐ FaceTime Video
- ☐ Doxy.me Video
- ☐ Other (please specify below)

Other:

CONCLUSION: Please respond with your opinion to the following questions:

	Strongly Agree	Agree	Neutral	Disagree	Strongly Disagree
When completing a telehealth visit, there are advantages to video visits over audio only.	○	○	○	○	○
The advantages of video visits outweigh the challenges of completing video visits.	○	○	○	○	○
As a provider, I have a desire to be able to schedule my own telehealth visits (video or audio only) in WakeOne.	○	○	○	○	○

If you would be willing to discuss your experiences with video visits, including stories of success, to help other providers, please enter your email. ______________________

Please provide us with any additional comments: ______________________
